# Spatial analysis and risk mapping of Crimean-Congo hemorrhagic fever (CCHF) in Sub-Saharan Africa

**DOI:** 10.1038/s41598-025-85873-8

**Published:** 2025-01-17

**Authors:** Abdoul Kader Ilboudo, Stephen Owambo Oloo, Jason Sircely, Ard M. Nijhof, Bernard Bett

**Affiliations:** 1 International Livestock Research Institute (ILRI), Human and Animal Health, Nairobi, Kenya; 2 Institute for Parasitology and Tropical Veterinary Medicine, Freie Universität Berlin, Berlin, Germany; 3 International Livestock Research Institute (ILRI), Policies Institutions and Livelihood, Nairobi, Kenya; 4 International Livestock Research Institute (ILRI), Sustainable Livestock Systems, Nairobi, Kenya; 5 Veterinary Centre for Resistance Research, Freie Universität Berlin, Berlin, Germany; 6 Departement biomédical et santé publique, Institut de Recherche en Sciences de la Santé (IRSS), Ouagadougou, Burkina Faso

**Keywords:** Spatial risk modeling, Crimean-Congo hemorrhagic fever, Sub-Saharan Africa, Ecological epidemiology, Ecological modelling, Environmental impact, Viral infection, Risk factors

## Abstract

Crimean Congo hemorrhagic fever (CCHF) is a re-emerging tick-borne zoonosis that is caused by CCHF virus (CCHFV). The geographical distribution of the disease and factors that influence its occurrence are poorly known. We analysed historical records on its outbreaks in various countries across the Sub-Saharan Africa (SSA) to identify hotspots and determine socioecological and demographic factors associated with these outbreaks. We used data from historical outbreaks that were reported between 1981 and 2022 in various countries in SSA. To develop a common framework for merging the outbreak data and potential explanatory variables, we generated a common shapefile that combined Level 2 administrative units in all the countries. Several climatic, environmental, socioecological data were obtained from on-line GIS databases and extracted using the shapefile. The data were analysed using an approximate Bayesian hierarchical model using the R-INLA package. The outcome was a Boolean variable which indicated whether an administrative unit in the shapefile was affected in a given year or not. A neighborhood structure was also generated and used to account for spatial autocorrelation in the analysis. The final model that was obtained from the analysis was used to build a CCHF risk map. A total of 54 CCHF outbreaks were compiled across 414 districts in nine SSA countries. Factors that were positively associated with CCHF outbreaks included human population density, land area under grassland, bare soil cover and shrub cover. Conversely, high precipitation during wet months, elevated mean temperature and slope had negative effects. The risk map generated shows that CCHF occurrence risk is higher in arid and semi-arid land (ASAL) of West Africa, the Sahelian region, Central Africa, and the Eastern and Southern Africa region. The analysis identified ecological and demographic factors that are associated with CCHF outbreaks in SSA. This finding suggests the need to improve surveillance for the disease especially in the grasslands where the human population is increasing.

**Supplementary Information:**

The online version contains supplementary material available at 10.1038/s41598-025-85873-8.

## Introduction

Crimean Congo hemorrhagic fever (CCHF) is a re-emerging vector-borne zoonotic disease caused by a tick-borne CCHF virus (CCHFV). The virus is prevalent throughout Africa, the Middle East, eastern Europe and parts of Asia, and it can infect a wide range of mammals and select bird species^1,2^. Unlike other arboviruses, CCHFV has evolved into multiple (seven) lineages (Asia 1, Asia 2, Africa 1, Africa 2, Africa 3, Europe 1, and Europe 2) probably to maintain its fitness in diverse environments, hosts and vectors^3^. In animals and birds, the virus is asymptomatic but in humans, a proportion of infections can result in clinical syndromes ranging from transient fevers to fatal hemorrhagic illness. Its case fatality rate in humans ranges between 10 and 50%^3,4^. Given its potential to cause pandemics, CCHF has been listed by the World Health Organization (WHO) as one of the diseases that deserves more attention^5^.

CCHF has a complex and poorly understood transmission cycle. The virus can be transmitted by several ixodid ticks, with some *Hyalomma*, *Amblyomma* and *Rhipicephalus *spp. being considered as efficient and natural vectors^3,6^. Many studies recognise *Hyalomma* spp. as the main vector and reservoir of the virus in arid and semi-arid areas where the prevailing climatic conditions favour the survival and development. The immature stages of most *Hyalomma*spp. feed on small mammals (lagomorphs) and birds while the adult stages feed on large mammals^7^. Given its ability to transmit the virus transovarially, transstadially and probably during co-feeding, infected *Hyalomma *ticks can infect the hosts it feeds on throughout all its development stages. Humans can get infected through the bite of an infected tick, contact with animal tissues with infection at the viraemic phase or contact with blood and infectious tissues from human patients at the acute phase of the disease^1^. CCHF outbreaks have also been observed in areas that not suitable for *Hyalomma* infestation. A recent study in Uganda indicated that *Rhipicephalus* and *Amblyomma *spp. could be responsible for the transmission of the virus in the affected areas^8^. This observation identifies the need for more work to characterize CCHFV in multiple ecological zones.

Many countries in Africa have a high risk of CCHF. Epidemics have been reported in a total of 20 countries in the continent^9^. Epidemiological studies that have been implemented in these countries have also observed high CCHFV seroprevalences in livestock^6^. Studies conducted in Mauritania^10^, Nigeria^11^, Mali^12^, the Gambia^13^, Uganda^14^, Kenya^15^and South Africa^16^, for example, indicate that cattle often show a high CCHFV seroprevalence ranging between 40 and 90%, while sheep and goats generally have a lower seroprevalence rate, ranging between 3–20%^17^. A few countries that have not reported human cases do have livestock that were exposed to CCHFV. In Malawi, for example, a cross sectional study reported a seroprevalence of 46.9% in cattle yet no human cases have been reported there^18^. In general, livestock exposure to CCHFV is often associated with advanced age and pastoral production systems or frequent movements. Risk factors for human infections include livestock farming, advanced age, and collecting or crushing ticks. Men are more likely to get exposed since they engage more in high risk occupations such as working in abattoirs or herding livestock^16,19^.

CCHF studies often focus on infection patterns of the virus in humans and livestock, and less on spatial distribution of the disease and its environmental risk factors^20^. Climatic and geographical factors influence the vector (tick) life cycle, vector-pathogen-host contacts and hence the CCHF risk levels. Changes in human and livestock demographics, livelihood practices and climate complicate the epidemiology of the disease^21,22^. Spatial and temporal clusters of CCHF were found in Greece^23^, Iran^24,25^and Bulgaria^26^. In addition, geographical factors such as land cover type, altitude, mean annual Enhanced Vegetation Index (EVI) land surface temperature and fragmentation of agricultural land were also found to be drivers of CCHF outbreak^27,28^. Recent studies found that average rainfall^22,29,30^, relative humidity^22,30^, average and maximum temperature^24,31^ are the climatic covariates most often associated with the emergence and maintenance of the disease in many regions. CCHF risk mapping studies focusing on the Sub-Saharan African region are limited and many of them, such as that by Messina et al.^29^. , have not included human and animal socio-demographic co-variates in addition to ecological ones. A good understanding on CCHF causal linkages at the environment /animal/ human interfaces in SSA would provide more insights for designing surveillance and control measures that are founded on the One Health framework.

We collated data on CCHF outbreaks in SSA and analysed them using spatial multivariable models to identify environmental and demographic factors that are associated with these outbreaks. We also used the models obtained from these analyses to develop a risk map.

## Methods

### CCHF occurrence data

CCHF outbreak data were collected from multiple data sources that include EM-DAT : a database created by the Centre for Research on the Epidemiology of Disasters (CRED), the Program for Monitoring Emerging Diseases (ProMED), World Health Organisation Disease Outbreak News (WHO-DON), the Center for Disease Control and Prevention (CDC) website, and the GIDEON (Global Infectious Diseases and Epidemiology Online Network) database. Outbreaks that were recorded between 01/01/1981 and 08/08/2022 were included in our database. Each confirmed case was considered as an outbreak as per the WHO definitions on hemorrhagic fevers in humans^32^. We also conducted a review of literature on PubMed, Google Scholar, Web of Science, Scopus and grey literature to capture more information on these outbreaks. Our search terms comprised Boolean combinations of ‘Crimean-Congo’, ‘Crimean’, ‘CCHF’, ‘CCHFV’, ‘Crimean-Congo haemorrhagic fever’, ‘Crimean haemorrhagic fever’ to search for CCHF outbreak occurrence including the American-English spelling variant for haemorrhagic (‘hemorrhagic’).

Each outbreak identified was transferred to the study database with its metadata that included the name of the area affected, name of the area from where the current outbreak originated, start and end dates, number of cases reported, the number of deaths observed, and the country affected. Each record was reviewed for its authenticity before it was appended with the rest of the data.

A shapefile that defines boundaries for level 2 administrative units (districts in most countries) in all the countries in the region was obtained from the Database of Global Administrative Areas (GADM)^33^. The shapefile was subsequently used to set up the primary data frame on which the outbreak data were merged. A Boolean variable “outbreak” was generated and used to identify districts that had been affected by CCHF. Each district with a reported outbreak was coded as 1 or 0 if not. To limit the effects of reporting bias while building the model, districts in countries that had not reported any outbreak were coded as “NA”. Their data were, therefore, not included in building the model but were used while forecasting risk to the entire spatial domain. Country names were defined based on the United Nations geoscheme definition of world geographic areas^34^. We added Sudan to our SSA countries regarding its particular location and to account for the role played by this country in CCHF circulation in the SSA country (cross-border events) (Appendices Table 3, Supplementary Material).

## Explanatory variables

**Fig. 1:**
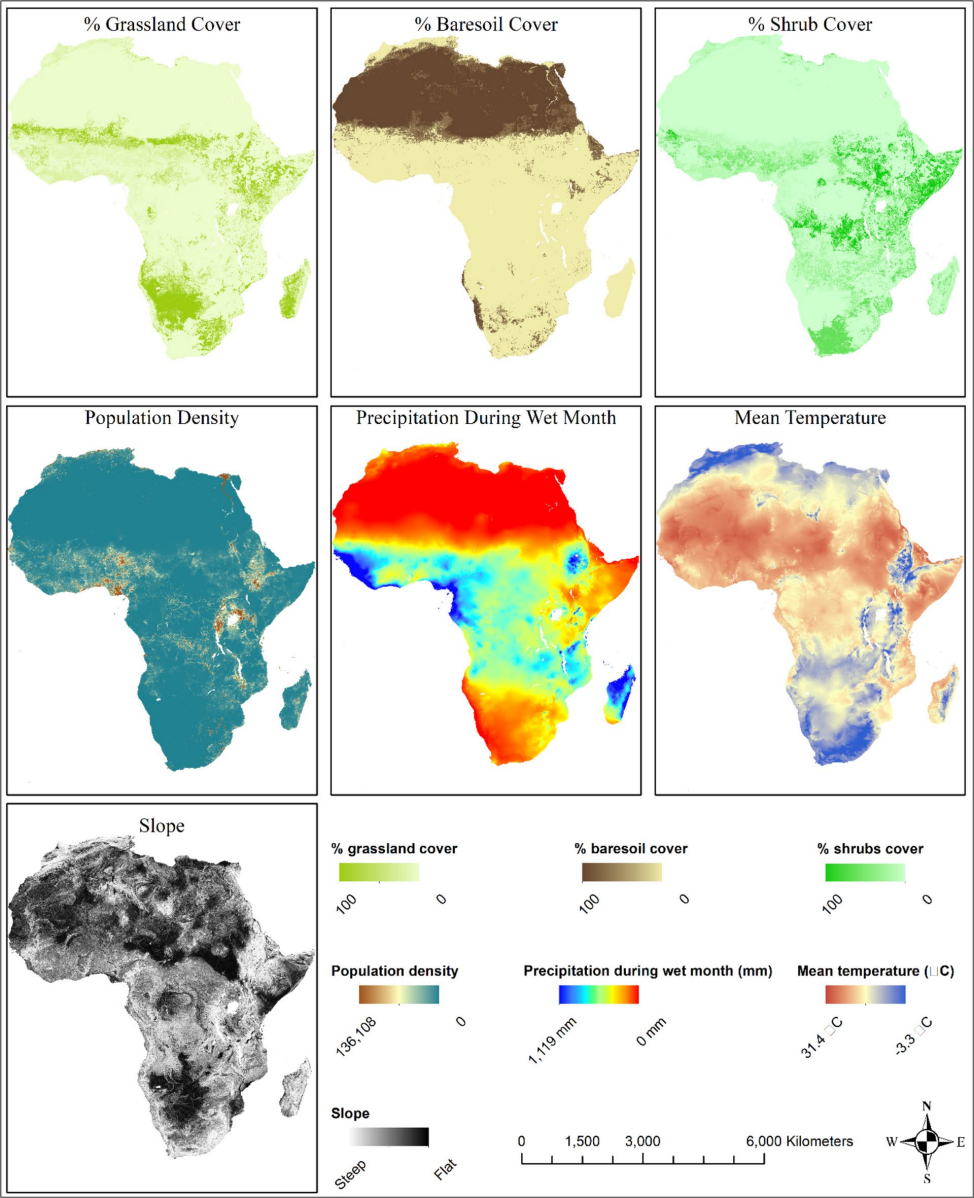
Spatial representation of main covariates used for the modelling. This figure was produced using ArcGIS 10.8 (c), the shapefile used for building the map was obtained from https://gadm.org/.

CCHF outbreaks are influenced by various explanatory variables. Understanding the interplay of these variables is essential for effective surveillance, prevention, and control of CCHF outbreaks. The explanatory variables included in our analysis were chosen based on biological evidence from the literature review on factors known or suspected to be associated with the emergence, maintenance and spread of the disease in general and more specifically in the context of the Sub-Saharan African region^1,3,20,35^. The identified factors allowed us to group the explanatory variables identified into three categories :1) climate variables, including the bioclimatic variables obtained from Worldclim^36^, 2) geographic variables that included the 2015 layer on time taken to travel to the nearest urban center, the spatial distribution of mammal species, the digital elevation model and slope, aridity index, Food and Agriculture Organisation (FAO) global landcover layers^37^, computed distance to the nearest national park or game reserve to the centroid of the districts, and wood cover layer, and 3) the demographic and socio-economic variables these included population density from Worldpop, livestock density and the percentage of births attended by specialists by country as a proxy for access to public health services (see Table II of the annexes)^38^ (Fig. 1). Apart from births attended by specialists and distance to the nearest national park or game reserve, the risk factor layers were downloaded from various sources as GEOTIFF files and were then extracted using the shapefile^39,41^. The livestock density data obtained were subsequently used to compute Tropical Livestock Units (TLU) for mammals and birds using Eqs. 1 and 2^42,40^. The average percentage of births attended by specialists was computed for the year 2000–2009 and 2010–2019. For each of the spatial layer covariates a mean for the admin 2 unit was extracted. The country mean of percentage of births attended by specialists was assigned to all the admin 2 units within the respectively countries^39^ (Appendices Table 4, Supplementary Material).

## Modelling the risk of CCHF occurrence

We utilized an approximate Bayesian hierarchical model to analyse the spatial distribution of CCHF outbreaks in the region. Besag-York-Mollié (BYM) model was used to model the spatial random effects due to its capacity to model spatially structured and unstructured components^43^.

Before we developed the model, we first checked for correlation among the predictors and selected only one variable from a set of highly correlated variables (a pair-wise correlation cutoff of 0.7 was used). The selected variables were then normalised using either log or square root transformation methods that best suited the data.

The model was developed using a combination of forward and backward variable selection techniques. At each step, 80% of the data was used to train the model and the remaining to test for prediction accuracy. The DIC (deviance information criterion), WAIC (Watanabe–Akaike information criterion), and the misclassification error were examined at each step. The analysis used the Integrated Nested Laplace Approximation (INLA) model, which was implemented using the R-INLA package^44^.

After model building, a Leave-One-Out Cross-Validation (LOOCV) was used for validation. After re-fitting the model with the remaining data, we used the updated model to predict the risk of a CCHF outbreak in the district that was left out. We repeated this LOOCV process for all 946 districts, generating a rich set of predictive performance metrics. The ultimate measure of our model’s predictive performance came in the form of the Area Under the Receiver Operating Characteristic (ROC) Curve (AUC), for evaluation of the accuracy of the predictive models.

## Model selection

Table 1 shows the steps that were taken to develop the final model (Model VII). While adding some variables increased the DIC (deviance information criterion), WAIC (Watanabe–Akaike information criterion), and the misclassification error, they were added to the model because they were significant. Any other variable that was added to Model VII was not significant; hence, we stopped at Model VII. During these steps, the mean spatial variation was reduced from 0.053 to 0.047.

Considering that a significant part of the outbreaks was reported from South Africa (20/54 outbreaks), we performed the same analysis without South Africa data. This analysis did not affect the results significantly; hence, it is not reported here.

**Table 1:** Model building.

Model	Significant Variables	DIC*	WAIC**	Miss-classification error
Model I	Sqrt***_grassland	796.82	820.68	0.0833
Model II	sqrt_grassland + log_baresoil_cover	836.48	904.06	0.0875
Model III	sqrt_grassland + log_baresoil_cover + sqrt_shrubs_cover	1163.11	1813.31	0.1042
Model IV	sqrt_grassland + log_baresoil_cover + sqrt_shrubs_cover + log_population	1140.01	2023.56	0.1042
Model V	sqrt_grassland + log_baresoil_cover + sqrt_shrubs_cover + log_population + wetmPrec	771.97	803.24	0.0958
Model VI	sqrt_grassland + log_baresoil_cover + sqrt_shrubs_cover + log_population + wetmPrec + meanTemp	789.24	840.45	0.0958
Model VII	sqrt_grassland + log_baresoil_cover + sqrt_shrubs_cover + log_population + wetmPrec + meanTemp + log_slope	791.67	854.53	0.0875

*DIC : deviance information criterion, **WAIC: Watanabe–Akaike information criterion.

***sqrt : square roots.

## Results

### Descriptive analysis

**Fig. 2:**
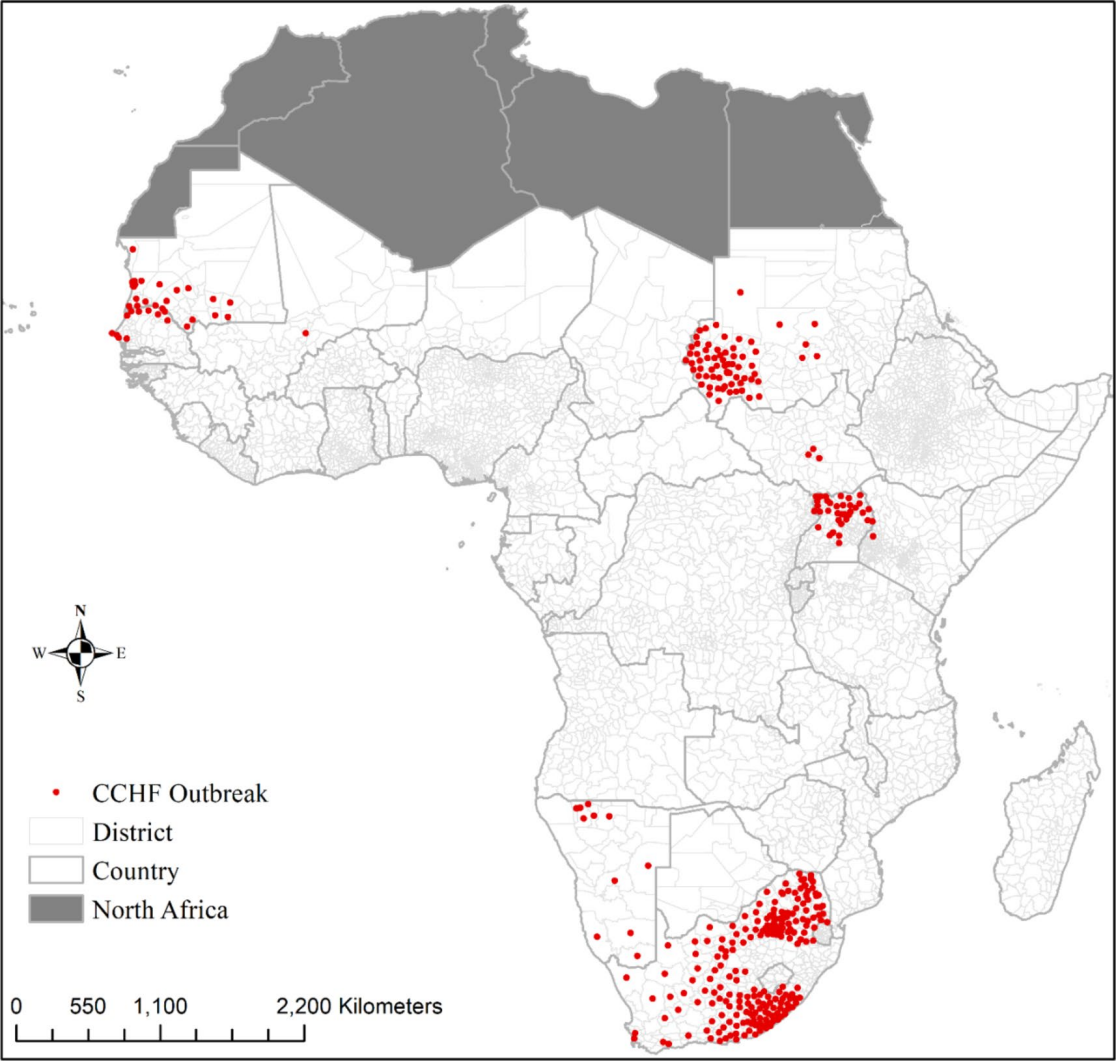
Geocoded CCHF outbreak location. This figure was produced using ArcGIS 10.8 (c), the shapefile used for building the map was obtained from https://gadm.org/.

A total of 54 CCHF outbreaks occurred in 414 Districts in nine (09) Sub-Saharan African countries between 1981 and 2022. More than 311 human cases were registered, with 52 fatal cases resulting in a case fatality rate of 16.7%. South Africa had 20 outbreaks, while only one was registered in Mali and Kenya. Spatial representation of CCHF cases on the map shows a high concentration in the Sahelo-Saharan region of West Africa (mainly Mauritania, Mali, and Senegal), East Africa (Sudan, South Sudan, and Uganda) and Southern Africa (mainly South Africa, and Namibia) (Fig. 2).

Human population density, grassland, bare soil cover, and shrub cover were positively associated with CCHF outbreak occurrence. Precipitation during wet months, mean temperature, and slopes were negatively related to the risk of CCHF outbreak (Table 2).

**Table 2:** Multivariable approximate bayesian hierarchical model of risk factors associated with CCHF outbreak in SSA (Model VII, table 1).

Covariates	Mean [95% CI]	SD*	Mode	Kld
***Intercept***	***12.4130 [1.4864***,*** 23.9420]***	***5.7165***	***12.0863***	***< 0.0001***
Square root grassland	0.4892 [0.2479, 0.7691]	0.1325	0.4692	< 0.0001
Log bare soil cover	0.9267 [0.4276,1.4898]	0.2702	0.8949	< 0.0001
Square root shrub cover	0.4718 [0.2034, 0.7706]	0.1442	0.4561	< 0.0001
Log population density	0.5377 [0.1785, 0.9309]	0.1915	0.5224	< 0.0001
Wet month precipitation	−0.1569 [−0.2921, −0.0298]	0.0668	−0.1526	< 0.0001
Mean temperature	−0.4762 [−0.8024, −0.1772]	0.1591	−0.4621	< 0.0001
Log slope	−2.1598 [−3.8047, −0.6136]	0.8122	−2.1065	< 0.0001

*SD: standard deviation ; Kld: Kullback-Leibler divergence (KLD).

The overall analysis of the risk map highlights a concentrated risk of CCHF outbreaks in the Sahelian zone of West Africa and East Southern Africa.

**Fig. 3:**
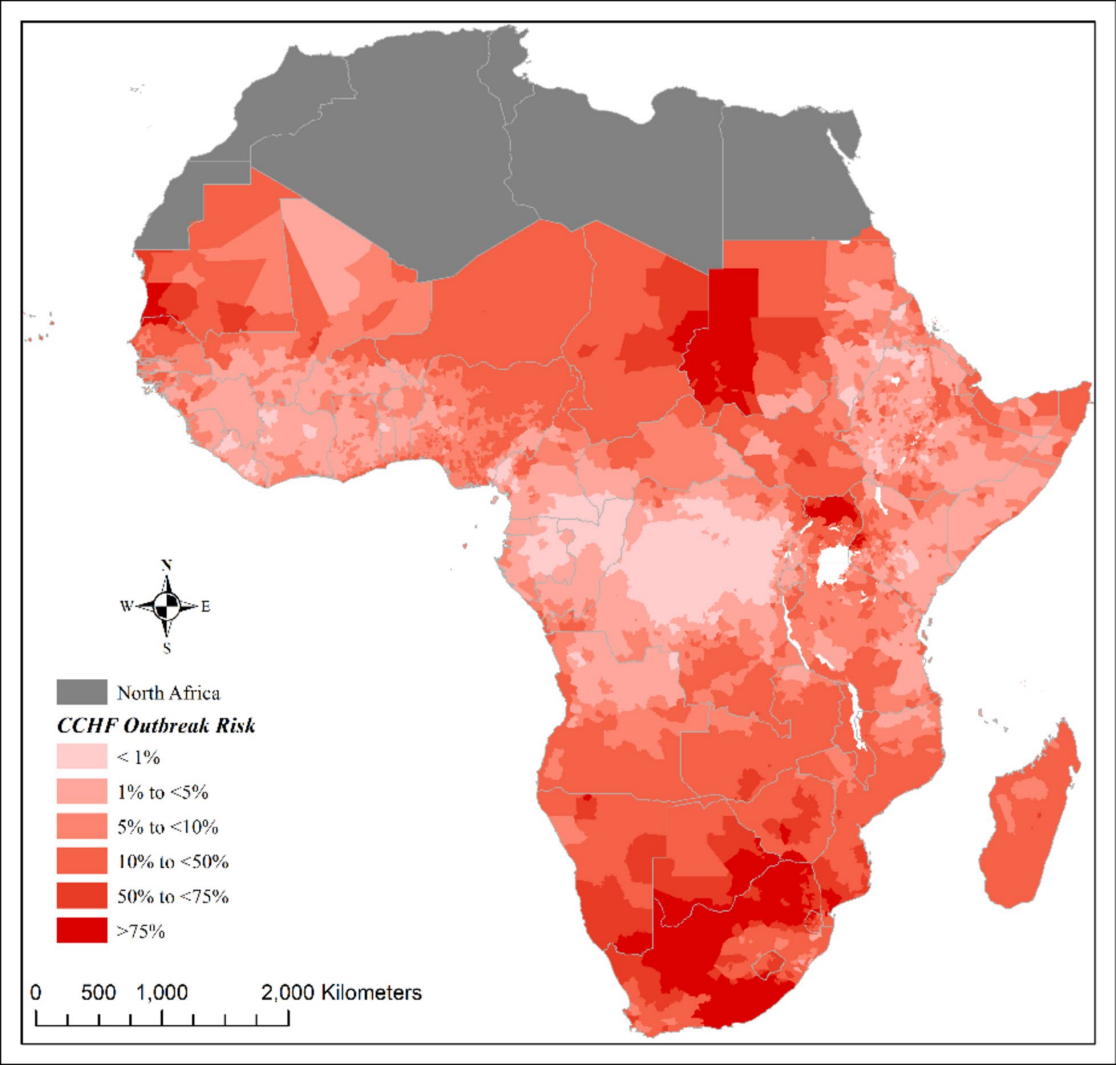
Risk map of CCHF occurrence in SSA. This figure was produced using ArcGIS 10.8 (c), the shapefile used for building the map was obtained from https://gadm.org/.

The probability of CCHF outbreak occurrence was over 75% in countries such as Mauritania, Senegal, and Mali in West Africa, South Sudan, Sudan, and Chad in Central Africa, Uganda and Kenya in East Africa, as well as South Africa, Botswana and Namibia in the South region. The risk is under 10% in West Africa along the Guinea Gulf and lower than 1% in most of the Democratic Republic of Congo, Republic of Congo, and Gabon in the Central Africa region (Fig. 3).

## Discussion

The study aims to determine the risk factors for CCHF and map the risks of potential epidemics, ultimately enabling us to establish a better surveillance system and control the disease. For this purpose, our analysis focused on the distribution of outbreaks in SAA and allowed us to build a risk map for CCHF outbreak occurrence in the region. While most studies have attempted to generate a CCHF risk map at the global-scale, our analysis focused on the SAA region to enable a more focused and higher resolution analyses. We were therefore able to include contextual analyses that are relevant for the region^29,45,46^. Proxy variables that captured the quality of the surveillance system and hence disease reporting biases such as accessibility to health care services, proximity to wildlife and the distribution of human population densities were included in the analyses. Our result shows that ecological factors, especially higher cover of grasslands, bare soil, and shrubs, were positively associated with CCHF outbreak occurrence. These results re-confirm previous works concluding that CCHF is primarily controlled by agroecological factors, with greatest prevalence in the arid and semi-arid land (ASAL), savanna grass and shrub habitats of *Hyalomma *spp. ticks, where rangeland livestock grazing is the predominant land use^20^. Grassland, bare soil, and shrub cover represent habitats conducive to tick survival and, proliferation and, habitation of diverse mammalian species including rodents therefore offering suitable microclimates for all tick life stages. Ideal land cover type combined with adequate microclimate is suitable for ticks population proliferation increasing the contact opportunities with humans and disease occurrence^47^. Seroprevalence and surveillance studies in Bulgaria, Turkey, and Iran emphasized the land cover type as a risk factor of CCHF presence^25–27^. In our model, the significance of land cover types in predicting the ecological niche for CCHF transmission to humans was greater than other variables. The same trends were found in country-wide spatial modeling studies in Turkey, India, and Uganda^28,31,48^, and in a global study^29^.

These findings on the ecological factors behind the onset of the disease should make it possible to determine the areas and conditions where surveillance needs to be stepped up. Several studies have also identified anthropogenic actions in the ecology, such as habitat fragmentation and land use, as drivers of CCHF^26,31^. Disease surveillance and prevention strategies such as vector control could be focused on the ASAL, savannah grass, and shrubs that are more suitable vector habitats.

Human population density is a risk factor for the spread of most epidemic diseases. In our model, the geographical distribution of the risk of CCHF is also a function of the population density. However, this linkage also needs to be put into perspective since the animal population density is also found to be a key factor in similar studies^23,27^. Relatively densely populated drylands subject to rangeland fragmentation are known to be an ideal environment for the emergence and spread of CCHF. The human population is generally positively correlated with domestic animal populations, which are the amplification hosts of CCHF. The correlation between human population density and the emergence of cases of CCHF in certain areas can simply be explained by their “attractiveness” to human activities and, hence, the exposure of residents and workers to tick bites by increasing the number of contact opportunities, and therefore, to the risk of CCHF^47^. The risk zones identified on the map suggest that drylands with higher human populations and more livestock are at higher risk of CCHF outbreaks than more arid, less populated areas.

However, in our model, the livestock population had no association with the occurrence of outbreaks. This could be an indication that the density of the livestock population may be more predictive of the seroprevalence of CCHF among animals than of the occurrence of human outbreaks. Similarly, Messina *et al.*, suggested that livestock reservoir abundance is more likely to influence the presence of the disease in an endemic area (seroprevalence) than to predict the overall risk of the disease^29^.

The risk map also confirms that forest zones are least associated with CCHF outbreaks; only a few cases of CCHF were notified in these regions. In addition, careful observation of this map shows “transitional risk zones” for CCHF outbreaks (probability: 10%-<50%) corresponding with land use transitional zones. Recent data showing the spread of the disease and/or its vector in previously unaffected areas could suggest that these transitional zones will be at greater risk of CCHF outbreaks in the coming years as a result of global warming^2,49,50^. The association between the existence of grazing land and the presence of CCHF can also be interpreted as an association between CCHF and the livestock population, particularly cattle and small ruminants bred in these pasture areas.

The negative association of outbreak risk with factors like precipitation during wet months, mean temperature, and slopes is likely correlative rather than causative. These factors may be useful in predicting CCHF risk due to phenomenological rather than mechanistic relationships. However, it is important to note that the presence of cases of CCHF is associated with climatic factors that are themselves linked to the presence of the vector. Precipitation, humidity, and temperature have been identified in several studies^22,24,30^. Therefore, favorable climatic and ecological conditions for the development and maintenance of the vector are necessary for epidemics to appear and be maintained over time. In addition, extreme weather conditions such as the one associated in our model may adversely affect tick activity and survival. Excessive rainfall during wet months can reduce tick abundance by drying out or flushing ticks from their habitats, while high temperatures can exceed the thermal limits for tick development and questing behavior^47,51^. To effectively model the geographical distribution of CCHF risk, it is crucial to understand the interactions between environmental and anthropogenic factors.

This study has potential limitations. We used secondary data from publicly reported outbreaks, which are highly affected by reporting bias. We may have missed CCHF cases that were never officially published or only circulated internally within governmental and international institutions. It is also important to note that countries have different surveillance capacities for human and veterinary diseases, which could explain the suspected under-notification of the disease. This could introduce bias in the final model and lead to misinterpretations. In addition, we used administrative level 2 areas to localize the CCHF cases. These borders were artificially created and do not necessarily reflect CCHF ecology, and neighboring areas can be ecologically different.

Additionally, predicting the occurrence of a CCHF epidemic seems rather complex, given the multiple interactions between host-animal-human and environment in this spillover event. Indeed, there are still many grey areas in the relationship between vector ticks, animals, and, above all, the interconnections between these entities and a rapidly changing environment, particularly in Sub-Saharan Africa.

## Conclusion

Identifying the hotspot of CCHF outbreak areas is essential in planning and implementing surveillance strategies. Robust human and veterinary disease surveillance is necessary to detect outbreaks in high-risk areas better. Our model shows that semi-arid and arid lands in SSA are the ideal zones for the occurrence of CCHF outbreaks as they may be the optimal tick habitat and meet most of the ecological drivers of the disease. Nevertheless, social factors such as population, livestock density, and human behavior in livestock-rearing practices play an important role in the occurrence of CCHF outbreaks. Our study could be a good starting point for a country-level risk map building in the high-risk region to better refine the disease patterns and implement surveillance and control strategies.

## Electronic supplementary material

Below is the link to the electronic supplementary material.

Supplementary Material 1

## Data Availability

The datasets used and/or analysed during the current study available from the corresponding author on reasonable request.

## References

[1] Ergönül, O. Crimean-Congo haemorrhagic fever. *Lancet Infect. Dis.* **6**, 203–214. 10.1016/S1473-3099(06)70435-2 (2006). 16554245 10.1016/S1473-3099(06)70435-2 PMC7185836

[2] Fillâtre, P., Revest, M. & Tattevin, P. Crimean-Congo hemorrhagic fever: an update. *Med. Mal Infect.* **49**, 574–585. 10.1016/j.medmal.2019.09.005 (2019). 31607406 10.1016/j.medmal.2019.09.005

[3] A Bente, D. et al. Crimean-Congo hemorrhagic fever: history, epidemiology, pathogenesis, clinical syndrome and genetic diversity. *Antiviral Res.* **100**, 159–189. 10.1016/j.antiviral.2013.07.006 (2013). 23906741 10.1016/j.antiviral.2013.07.006

[4] Ergonul, O. Treatment of Crimean-Congo hemorrhagic fever. *Antiviral Res.* **78**, 125–131 (2008). 18096251 10.1016/j.antiviral.2007.11.002

[5] World Health Organisation. Crimean-Congo Haemorragic fever fact-sheets. (2022). https://www.who.int/news-room/fact-sheets/detail/crimean-congo-haemorrhagic-fever

[6] Belobo, J. T. E. et al. Worldwide epidemiology of Crimean-Congo Hemorrhagic Fever Virus in humans, ticks and other animal species, a systematic review and meta-analysis. *PLoS Negl. Trop. Dis.* **15**, e0009299. 10.1371/journal.pntd.0009299 (2021). 33886556 10.1371/journal.pntd.0009299 PMC8096040

[7] Nasirian, H. Ticks infected with crimean-Congo hemorrhagic fever virus (CCHFV): a decision approach systematic review and meta-analysis regarding their role as vectors. *Travel Med. Infect. Dis.* **47**, 102309. 10.1016/j.tmaid.2022.102309 (2022). 35318129 10.1016/j.tmaid.2022.102309

[8] Lule, S. A. et al. Widespread exposure to Crimean-Congo haemorrhagic fever in Uganda might be driven by transmission from Rhipicephalus ticks: evidence from cross-sectional and modelling studies. *J. Infect.* **85**, 683–692 10.1016/j.jinf.2022.09.016 (2022). 10.1016/j.jinf.2022.09.016 36152736

[9] Temur, A. I., Kuhn, J. H., Pecor, D. B., Apanaskevich, D. A. & Keshtkar-Jahromi, M. Epidemiology of Crimean-Congo Hemorrhagic Fever (CCHF) in Africa—underestimated for decades. *Am. J. Trop. Med. Hyg.* **104**, 1978–1990. 10.4269/ajtmh.20-1413 (2021). 33900999 10.4269/ajtmh.20-1413 PMC8176481

[10] Sas, M. A. et al. Crimean-Congo Hemorrhagic Fever Virus-specific antibody detection in cattle in Mauritania. *Vector Borne Zoonotic Dis. (Larchmont N Y)*. **17**, 582–587. 10.1089/vbz.2016.2084 (2017). 10.1089/vbz.2016.2084 28605299

[11] Dzikwi-Emennaa, A. A. et al. Detection of Crimean-Congo hemorrhagic fever virus antibodies in cattle in Plateau State, Nigeria. *Viruses* **14**, 2618 (2022). 10.3390/v14122618 PMC9787510 36560622

[12] Maiga, O. et al. Serosurvey of Crimean-Congo hemorrhagic fever virus in Cattle, Mali, West Africa. *Am. J. Trop. Med. Hyg.* **96**, 1341–1345. 10.4269/ajtmh.16-0818 (2017). 10.4269/ajtmh.16-0818 PMC5462568 28719259

[13] Matthews, J., Secka, A., McVey, D. S., Dodd, K. A. & Faburay, B. Serological Prevalence of Crimean–Congo Hemorrhagic Fever Virus Infection in Small Ruminants and Cattle in The Gambia. *Pathogens* 12, 749%@ 2076 – 0817 (2023). 10.3390/pathogens12060749 PMC10302318 37375439

[14] Atim, S. A. et al. Prevalence of Crimean-Congo haemorrhagic fever in livestock following a confirmed human case in Lyantonde district, Uganda. *Parasit. Vectors*. **16**, 71756–73305 (2023). 10.1186/s13071-022-05588-x PMC9824997 36611216

[15] Omoga, D. C. A. et al. Transmission Dynamics of Crimean–Congo Haemorrhagic Fever Virus (CCHFV): Evidence of Circulation in Humans, Livestock, and Rodents in Diverse Ecologies in Kenya. *Viruses* 15, 1891%@ 1999–4915 (2023). 10.3390/v15091891 PMC10535211 37766297

[16] Msimang, V. et al. Risk factors associated with exposure to Crimean-Congo haemorrhagic fever virus in animal workers and cattle, and molecular detection in ticks, South Africa. *PLoS Negl. Trop. Dis.* **15**, e0009384. 10.1371/journal.pntd.0009384 (2021). 34048430 10.1371/journal.pntd.0009384 PMC8162673

[17] Spengler, J. R., Bergeron, E. & Rollin, P. E. Seroepidemiological studies of Crimean-Congo Hemorrhagic Fever Virus in domestic and wild animals. *PLoS Negl. Trop. Dis.* **10**, e0004210. 10.1371/journal.pntd.0004210 (2016). 26741652 10.1371/journal.pntd.0004210 PMC4704823

[18] Phonera, M. et al. (ed, C.) Seroprevalence and risk factors of Crimean-Congo hemorrhagic fever in cattle of smallholder farmers in Central Malawi. *Pathogens* **10** 1613–2076 (2021). 34959568 10.3390/pathogens10121613 PMC8709441

[19] Akuffo, R. et al. Crimean-Congo hemorrhagic fever virus in livestock ticks and animal handler seroprevalence at an abattoir in Ghana. *BMC Infect. Dis.* **16**, 324. 10.1186/s12879-016-1660-6 (2016). 27392037 10.1186/s12879-016-1660-6 PMC4939019

[20] Sorvillo, T. E. et al. Towards a sustainable one health approach to crimean–congo hemorrhagic fever prevention: Focus areas and gaps in knowledge. *Tropical Medicine and Infectious Disease* 5, 113%@ 2414–6366 (2020). 10.3390/tropicalmed5030113 PMC7558268 32645889

[21] Ansari, H. et al. Crimean-Congo hemorrhagic fever and its relationship with climate factors in southeast Iran: a 13-year experience. *J. Infect. Developing Ctries.* **8**, 749–7571972 (2014). 10.3855/jidc.4020 24916874

[22] Duygu, F., Sari, T., Kaya, T., Tavsan, O. & Naci, M. The relationship between Crimean-Congo hemorrhagic fever and climate: does climate affect the number of patients? *Acta Clin. Croatica*. **57**, 443 (2018). 10.20471/acc.2018.57.03.06 PMC6536269 31168176

[23] Papa, A., Sidira, P. & Tsatsaris, A. Spatial cluster analysis of Crimean-Congo hemorrhagic fever virus seroprevalence in humans, Greece. *Parasite Epidemiol. Control*. **1**, 211–218. 10.1016/j.parepi.2016.08.002 (2016). 29988220 10.1016/j.parepi.2016.08.002 PMC5991861

[24] Ahmadkhani, M., Alesheikh, A. A., Khakifirouz, S. & Salehi-Vaziri, M. Space-time epidemiology of Crimean-Congo hemorrhagic fever (CCHF) in Iran. *Ticks Tick. Borne Dis.* **9**, 207–216 10.1016/j.ttbdis.2017.09.006 (2018). 10.1016/j.ttbdis.2017.09.006 28943247

[25] Mostafavi, E., Haghdoost, A., Khakifirouz, S. & Chinikar, S. Spatial analysis of Crimean Congo hemorrhagic fever in Iran. *Am. J. Trop. Med. Hyg.* **89**, 1135–1141. https://doi.org/10.4269/ajtmh.12–0509 (2013). 10.4269/ajtmh.12-0509 PMC3854891 24166038

[26] Vescio, F. M. et al. Environmental correlates of Crimean-Congo haemorrhagic fever incidence in Bulgaria. *BMC Public. Health*. **12**, 1116. 10.1186/1471-2458-12-1116 (2012). 23270399 10.1186/1471-2458-12-1116 PMC3547815

[27] Aker, S., Akinci, H., Kilicoglu, C. & Leblebicioglu, H. The geographic distribution of cases of Crimean-Congo hemorrhagic fever: Kastamonu, Turkey. *Ticks Tick. Borne Dis.* **6**, 730–736. 10.1016/j.ttbdis.2015.06.008 (2015). 26139033 10.1016/j.ttbdis.2015.06.008

[28] Estrada-Pena, A. et al. Modeling the spatial distribution of crimean-congo hemorrhagic fever outbreaks in Turkey. *Vector Borne Zoonotic Dis.* **7**, 667–678. 10.1089/vbz.2007.0134 (2007). 18047397 10.1089/vbz.2007.0134

[29] Messina, J. P. et al. The global distribution of Crimean-Congo hemorrhagic fever. *Trans. R. Soc. Trop. Med. Hyg.* **109**, 503–513. 10.1093/trstmh/trv050 (2015). 26142451 10.1093/trstmh/trv050 PMC4501401

[30] Nili, S., Khanjani, N., Jahani, Y. & Bakhtiari, B. The effect of climate variables on the incidence of Crimean Congo Hemorrhagic Fever (CCHF) in Zahedan, Iran. *BMC Public. Health* **20**, 1893 10.1186/s12889-020-09989-4 (2020). 10.1186/s12889-020-09989-4 PMC7726875 33298021

[31] Chanda, M. M. et al. Quantifying the influence of climate, host and change in land-use patterns on occurrence of Crimean Congo Hemorrhagic Fever (CCHF) and development of spatial risk map for India. *One Health*. **17** https://doi.org/10.1016/j.onehlt.2023.100609 (2023). 10.1016/j.onehlt.2023.100609 PMC10424211 37583365

[32] Mofleh, J. & Ahmad, Z. Crimean-Congo haemorrhagic fever outbreak investigation in the Western Region of Afghanistan in 2008. *East. Mediterr. Health J.* **18**, 522–526. 10.26719/2012.18.5.522 (2012). 22764441 10.26719/2012.18.5.522

[33] GADM database of global administrative areas (V 3.6) (2018-2022). https://gadm.org/download_world36.html

[34] United Nations Statistics Division Methodology. Standard country or area codes for statistical use (M49) (2020). https://unstats.un.org/unsd/methodology/m49/

[35] Kuehnert, P. et al. Defining the social determinants of health for nursing action to achieve health equity: a consensus paper from the American Academy of Nursing. *Nurs. Outlook*. **70**, 10–27. 10.1016/j.outlook.2021.08.003 (2022). 34629190 10.1016/j.outlook.2021.08.003

[36] Fick, S. E. & Hijmans, R. J. WorldClim 2: new 1-km spatial resolution climate surfaces for global land areas. *Int. J. Climatol.* **37**, 4302–43150899 (2017).

[37] Latham, J., Cumani, R., Rosati, I. & Bloise, M. Global land cover share (GLC-SHARE) database beta-release version 1.0-2014. *FAO: Rome Italy* **29**. https://www.fao.org/uploads/media/glc-share-doc.pdf (2014).

[38] WorldPop Population density data, (2023). https://hub.worldpop.org/.

[39] World Bank Gender Data Portal. Births attended by skilled health staff (% of total) (2023). https://genderdata.worldbank.org/indicators/sh-sta-brtc-zs

[40] United Nations Environment Programme World Conservation Monitoring Centre (UNEP-WCMC). *International Union for Conservation of Nature (IUCN). The World Database on Protected Areas (WDPA), the Global Database on Protected Areas Management Effectiveness* (GD-PAME), 2019).

[41] Rothman-Ostrow, P., Gilbert, W. & Rushton, J. Tropical livestock units: re-evaluating a methodology. *Front. Veterinary Sci. ***7**, 556788. 10.3389/fvets.2020.556788 (2020). 10.3389/fvets.2020.556788 PMC7714756 33330685

[42] Chilonda, P. & Otte, J. Indicators to monitor trends in livestock production at national, regional and international levels.

[43] Besag, J., York, J. & Mollié, A. Bayesian image restoration, with two applications in spatial statistics. *Ann. Inst. Stat. Math.* **43**, 1–200020 (1991).

[44] Bachl, F. E., Lindgren, F., Borchers, D. L. & Simpson, D. & Scott-Hayward, L. Inlabru: spatial inference using integrated nested Laplace approximation. *R Package Version* 2. http://cran.nexr.com/web/packages/inlabru/inlabru.pdf (2018).

[45] Messina, J. & Wint, W. The spatial distribution of Crimean-Congo haemorrhagic fever in Europe and neighbouring areas: December 2023. (2023). 10.3390/insects14090771 PMC10532370 37754739

[46] Okely, M., Anan, R., Gad-Allah, S. & Samy, A. M. Mapping the environmental suitability of etiological agent and tick vectors of Crimean-Congo hemorrhagic fever. *Acta Trop.* **203**, 105319. 10.1016/j.actatropica.2019.105319 (2020). 31874130 10.1016/j.actatropica.2019.105319

[47] Estrada-Pena, A. & de la Fuente, J. The ecology of ticks and epidemiology of tick-borne viral diseases. *Antiviral Res.* **108**, 104–128. 10.1016/j.antiviral.2014.05.016 (2014). 24925264 10.1016/j.antiviral.2014.05.016

[48] Telford, C., Nyakarahuka, L., Waller, L., Kitron, U. & Shoemaker, T. Spatial prediction of Crimean Congo hemorrhagic fever virus seroprevalence among livestock in Uganda. *One Health*. **17**, 100576. 10.1016/j.onehlt.2023.100576 (2023). 38024282 10.1016/j.onehlt.2023.100576 PMC10665170

[49] Bonnet, S. I. et al. The control of Hyalomma ticks, vectors of the Crimean-Congo hemorrhagic fever virus: where are we now and where are we going? *PLoS Negl. Trop. Dis.* **16**, e0010846. 10.1371/journal.pntd.0010846 (2022). 36395110 10.1371/journal.pntd.0010846 PMC9671348

[50] Bah, M. T. et al. The Crimean-Congo haemorrhagic fever tick vector Hyalomma marginatum in the south of France: modelling its distribution and determination of factors influencing its establishment in a newly invaded area. *Transbound. Emerg. Dis.* **69**, e2351–e2365. 10.1111/tbed.14578 (2022). 35511405 10.1111/tbed.14578 PMC9790221

[51] Diuk-Wasser, M. A., VanAcker, M. C. & Fernandez, M. P. Impact of Land Use Changes and Habitat Fragmentation on the eco-epidemiology of Tick-Borne diseases. *J. Med. Entomol.* **58**, 1546–1564. 10.1093/jme/tjaa209 (2021). 33095859 10.1093/jme/tjaa209

[52] Centers for Diseases Control. Morbidity and mortality weekly report. International Notes Crimean-Congo Hemorrhagic Fever. *Repub. South. Afr.* **34**(7), 99–101. https://www.cdc.gov/mmwr/preview/mmwrhtml/00000489.htm (1985). 3918252

[53] Gonzalez, J. P., LeGuenno, B., Guillaud, M. & Wilson, M. L. A fatal case of Crimean-Congo haemorrhagic fever in Mauritania: virological and serological evidence suggesting epidemic transmission. *Trans. R Soc. Trop. Med. Hyg.* **84**, 573–576. 10.1016/0035-9203(90)90045-g (1990). 2128671 10.1016/0035-9203(90)90045-g

[54] World Health Organisation. Crimean-Congo Haemorrhagic Fever in South Africa (1996). https://www.who.int/emergencies/disease-outbreak-news/item/1996_11_05-en

[55] Dunster, L. et al. First documentation of human crimean-Congo hemorrhagic fever, Kenya. *Emerg. Infect. Dis.* **8**, 1005 (2002). 12194785 10.3201/eid0809.010510 PMC2732535

[56] Nabeth, P. et al. Crimean-Congo hemorrhagic fever, Mauritania. *Emerg. Infect. Dis.* **10**, 2143–2149. 10.3201/eid1012.040535 (2004). 15663851 10.3201/eid1012.040535 PMC3323392

[57] Nabeth, P., Thior, M., Faye, O. & Simon, F. Human crimean-Congo Hemorrhagic Fever, Sénégal. *Emerg. Infect. Disease J.* **10**, 1881. 10.3201/eid1010.040586 (2004). 10.3201/eid1010.040586 PMC3323271 15565746

[58] Tarantola, A., Nabeth, P., Tattevin, P., Michelet, C. & Zeller, H. Lookback exercise with imported Crimean-Congo hemorrhagic fever, Senegal and France. *Emerg. Infect. Dis.* **12**, 1424–1426. 10.3201/eid1209.060002 (2006). 17073094 10.3201/eid1209.060002 PMC3294739

[59] Aradaib, I. E. et al. Nosocomial outbreak of Crimean-Congo hemorrhagic fever, Sudan. *Emerg. Infect. Dis.* **16**, 837–839. 10.3201/eid1605.091815 (2010). 20409377 10.3201/eid1605.091815 PMC2954009

[60] Aradaib, I. E. et al. Multiple crimean-Congo hemorrhagic fever virus strains are associated with disease outbreaks in Sudan, 2008–2009. *PLoS Negl. Trop. Dis.* **5**, e1159. 10.1371/journal.pntd.0001159 (2011). 21655310 10.1371/journal.pntd.0001159 PMC3104971

[61] Balinandi, S. et al. Investigation of an isolated case of human crimean-Congo hemorrhagic fever in Central Uganda, 2015. *Int. J. Infect. Dis.* **68**, 88–93 10.1016/j.ijid.2018.01.013 (2018). 10.1016/j.ijid.2018.01.013 PMC5893389 29382607

[62] Bob, N. S. et al. Detection of the Northeastern African Rift Valley Fever Virus Lineage During the 2015 Outbreak in Mauritania. Open Forum Infect. Dis. 4, ofx087 (2017) 10.1093/ofid/ofx087 PMC5473438 28638845

[63] Boushab, B. M., Kelly, M., Kébé, H., Bollahi, M. A. & Basco, L. K. Crimean-Congo Hemorrhagic Fever, Mauritania. *Emerg. Infect. Dis.* **26**, 817–818. 10.3201/eid2604.191292 (2020). 32187505 10.3201/eid2604.191292 PMC7101092

[64] Dieng, I. et al. Detection of Crimean Congo haemorrhagic fever virus in North-eastern Senegal, Bokidiawé 2019. *Emerg. Microbes Infect.* **9**, 2485–2487. 10.1080/22221751.2020.1847605 (2020). 33161829 10.1080/22221751.2020.1847605 PMC7717587

[65] Samaké, D. et al. Crimean-Congo Hemorrhagic Fever in Mopti: epidemiological, clinical and diagnostic aspects. *Innovative J. Med. Health Sci.* vol. 11, no. 01 (Jan- Feb) (2021).

[66] Outbreak News Today. South Africa reports 1st Crimean-Congo hemorrhagic fever case of 2021%U (2021). https://outbreaknewstoday.com/south-africa-reports-1st-crimean-congo-hemorrhagic-fever-case-of-2021

[67] Outbreak News Today. Crimean-Congo hemorrhagic fever cases reported in Senegal (2022). https://outbreaknewstoday.com/crimean-congo-hemorrhagic-fever-cases-reported-in-senegal-10604/(

[68] Bower, H. et al. Detection of Crimean-Congo Haemorrhagic Fever cases in a severe undifferentiated febrile illness outbreak in the Federal Republic of Sudan: a retrospective epidemiological and diagnostic cohort study. *PLoS Negl. Trop. Dis.* **13**, e0007571. 10.1371/journal.pntd.0007571 (2019). 31291242 10.1371/journal.pntd.0007571 PMC6645580

[69] Kizito, S. et al. Notes from the field: Crimean-Congo Hemorrhagic Fever outbreak - central Uganda, August-September 2017. *MMWR Morb Mortal. Wkly. Rep.* **67**, 646–647. 10.15585/mmwr.mm6722a6 (2018). 29879093 10.15585/mmwr.mm6722a6 PMC5991810

[70] Weiss, D. J. et al. A global map of travel time to cities to assess inequalities in accessibility in 2015. *Nature* **553**, 333–336. 10.1038/nature25181 (2018). 29320477 10.1038/nature25181

[71] International Union for Conservation of Nature, I. & Center for International Earth Science Information Network. C. C. U. (NASA Socioeconomic Data and Applications Center (SEDAC) %U. https://doi.org/10.7927/H4N014G5%8 20231127, Palisades, New York (2015).

[72] Baz-Flores, S. et al. Animal exposure model for Mapping Crimean-Congo Hemorrhagic Fever Virus Emergence Risk. *Emerg. Infect. Dis.* **30**, 672–680. 10.3201/eid3004.221604 (2024). 38526057 10.3201/eid3004.221604 PMC10977842

[73] Trabucco, A. & Zomer, R. J. Global aridity index and potential evapotranspiration (ET0) climate database v2. *CGIAR Consort Spat. Inf.* **10**, m9 (2018).

[74] El Ghassem, A. et al. Risk factors associated with crimean-Congo hemorrhagic fever virus circulation among human, livestock and ticks in Mauritania through a one health retrospective study. *BMC Infect. Dis.* **23**, 764. 10.1186/s12879-023-08779-8 (2023). 37932678 10.1186/s12879-023-08779-8 PMC10626674

[75] Fanelli, A. et al. Epidemic intelligence data of Crimean-Congo haemorrhagic fever, European Region, 2012 to 2022: a new opportunity for risk mapping of neglected diseases. *Euro. Surveill*. **28**. 10.2807/1560-7917.ES.2023.28.16.2200542 (2023). 10.2807/1560-7917.ES.2023.28.16.2200542 PMC10283452 37078883

[76] Farr, T. G. et al. The shuttle radar topography mission. *Rev. Geophys*. **45**, RG2004. 10.1029/2005RG000183 (2007).

[77] Gilbert, M. et al. Global distribution data for cattle, buffaloes, horses, sheep, goats, pigs, chickens and ducks in 2010. *Sci. Data*. **5**, 180227. 10.1038/sdata.2018.227 (2018). 30375994 10.1038/sdata.2018.227 PMC6207061

[78] Kahiu, M. N. & Hanan, N. P. Fire in sub-saharan Africa: the fuel, cure and connectivity hypothesis. *Glob. Ecol. Biogeogr.* **27**, 946–9571466 (2018).

[79] Blair, P. W. et al. An emerging Biothreat: Crimean-Congo Hemorrhagic Fever Virus in Southern and Western Asia. *Am. J. Trop. Med. Hyg.* **100**, 16–23. 10.4269/ajtmh.18-0553 (2019). 30652673 10.4269/ajtmh.18-0553 PMC6335890

