# Author Correction: Spatial analysis and risk mapping of Crimean-Congo hemorrhagic fever (CCHF) in Sub-Saharan Africa

**DOI:** 10.1038/s41598-025-93607-z

**Published:** 2025-03-24

**Authors:** Abdoul Kader Ilboudo, Stephen Owambo Oloo, Jason Sircely, Ard M. Nijhof, Bernard Bett

**Affiliations:** 1 International Livestock Research Institute (ILRI), Human and Animal Health, Nairobi, Kenya; 2 Institute for Parasitology and Tropical Veterinary Medicine, Freie Universität Berlin, Berlin, Germany; 3 International Livestock Research Institute (ILRI), Policies Institutions and Livelihood, Nairobi, Kenya; 4 International Livestock Research Institute (ILRI), Sustainable Livestock Systems, Nairobi, Kenya; 5 Veterinary Centre for Resistance Research, Freie Universität Berlin, Berlin, Germany; 6 Departement biomédical et santé publique, Institut de Recherche en Sciences de la Santé (IRSS), Ouagadougou, Burkina Faso

Correction to: *Scientific Reports* 10.1038/s41598-025-85873-8, published online 17 January 2025

The original version of this Article contained errors in affiliations 1, 3 and 4, where the city and country were incorrectly given as ‘Berlin, Germany’. The correct affiliations are listed below:

1 International Livestock Research Institute (ILRI), Human and Animal Health, Nairobi, Kenya.

3 International Livestock Research Institute (ILRI), Policies Institutions and Livelihood, Nairobi, Kenya.

4 International Livestock Research Institute (ILRI), Sustainable Livestock Systems, Nairobi, Kenya.

The original Article has been corrected.

