# Supplementary material for: Spatial analysis and risk mapping of Crimean-Congo hemorrhagic fever (CCHF) in Sub-Saharan Africa
Source: Sci Rep. 2025 Jan 17;15:2292. doi: 10.1038/s41598-025-85873-8 (PMC11742035; doi:10.1038/s41598-025-85873-8)
Supplement: Supplementary file 1 — Supplementary Material 1 [file 41598_2025_85873_MOESM1_ESM.docx]

APPENDICES:

**Table 3**: Description of CCHF outbreaks reported in SSA from 1981 to 2022

| **Outbreak Number** | **country** | **Outbreak location** | **year** | **first case date** | **number cases** | **Number of fatal cases** | **case fatality rate (%)** | **cross_border events** | **previous evidence of CCHF circulation** | **Data source/ reference** |
| --- | --- | --- | --- | --- | --- | --- | --- | --- | --- | --- |
| 1 | South Africa | Vredenburg district | 1984 | 28/08/1984 | 8 | 2 | 25.0 | --- | Yes | ^52^ |
| 2 | Mauritania | south-western | 1988 | 01/05/1988 | 8 | 1 | 12.5 | no | yes | ^53^ |
| 3 | South Africa | Oudtshoorn, Western Cape Province | 1996 | 04/11/1996 | 32 | 1 | 3.1 | no | no | ^54^ |
| 4 | South Africa | Northern Cape Povince | 1998 | 28/02/1998 | 1 | 1 | 100.0 | no | yes | International Society for Infectious Diseases, ProMED  Archive  number: 19980317.0494  [Promed Post - ProMED-mail (promedmail.org)](https://promedmail.org/promed-post/?id=19980317.0494) |
| 5 | South Africa | Eastern Cape | 2000 | 03/02/2000 | 1 | 1 | 100.0 | no | yes | International Society for Infectious Diseases, .ProMED  Archive  number: 20000203.0161  [Promed Post - ProMED-mail (promedmail.org)](https://promedmail.org/promed-post/?id=20000203.0161) |
| 6 | Kenya | western | 2000 | 21/10/2000 | 1 | 1 | 100.0 | no | yes | ^55^ |
| 7 | Namibia | Gobabis | 2001 | 25/01/2001 | 1 | 1 | 100.0 | no | yes | International Society for Infectious Diseases, .ProMED  Archive  number: 20010125.0179  [Promed Post - ProMED-mail (promedmail.org)](https://promedmail.org/promed-post/?id=20010125.0179) |
| 8 | South Africa | Cape town | 2001 | 04/02/2001 | 1 | 0 | 0.0 | no | yes | International Society for Infectious Diseases, .ProMED  Archive  number: 20010214.0295  [Promed Post - ProMED-mail (promedmail.org)](https://promedmail.org/promed-post/?id=20010214.0295) |
| 9 | Namibia | Katutura | 2002 | 16/01/2002 | 1 | 1 | 100.0 | no | yes | International Society for Infectious Diseases, .ProMED  Archive  number: 20020120.3327  [Promed Post - ProMED-mail (promedmail.org)](https://promedmail.org/promed-post/?id=20020120.3327) |
| 10 | South Africa | Northern Cape | 2002 | --- | 1 | -- | 0.0 | -- | -- | Gideon online  [Global Infectious Diseases and Epidemiology Network \| GIDEON (gideononline.com)](https://www.gideononline.com/) |
| 11 | Mauritania | Nouakchott( trarza, Brakna, Hodh Gharbi) | 2003 | 17/02/2003 | 38 | 6 | 15.8 | no | yes | ^56^ |
| 12 | Senegal | Popenguine District | 2003 | 26/01/2003 | 1 | 0 | 0.0 | no | yes | ^57^ |
| 13 | Senegal | Sally | 2004 | 04/11/2004 | 2 | 1 | 50.0 | yes(France) | yes | ^58^ |
| 14 | Mauritania | Dar Naim | 2006 | --- | 1 |  | 0.0 | -- | -- | Gideon online  [Global Infectious Diseases and Epidemiology Network \| GIDEON (gideononline.com)](https://www.gideononline.com/) |
| 15 | South Africa | Swellendam and Heidelberg | 2006 | ----- | 1 |  | 0.0 | -- | -- | Gideon online  [Global Infectious Diseases and Epidemiology Network \| GIDEON (gideononline.com)](https://www.gideononline.com/) |
| 16 | South Africa | Petrusburg area in the Free State Province | 2006 | 19/01/2006 | 1 | 1 | 100.0 | no | yes | International Society for Infectious Diseases, .ProMED  Archive  number: 20060119.0175  [Promed Post - ProMED-mail (promedmail.org)](https://promedmail.org/promed-post/?id=20060119.0175) |
| 17 | Sudan | Kordufan | 2008 | 10/1/2008 | 8 | 6 | 75.0 | no | yes | ^59^ |
| 18 | South Africa | Eastern Cape (Adelaide) | 2008 | 12/07/2008 | 1 | 1 | 100.0 | no | yes | International Society for Infectious Diseases, .ProMED  Archive  number: 20080716.2162  [Promed Post - ProMED-mail (promedmail.org)](https://promedmail.org/promed-post/?id=20080716.2162) |
| 19 | Mauritania | Arafat, Moujeria | 2009 |  | 2 |  | 0.0 | -- | -- | Gideon online  [Global Infectious Diseases and Epidemiology Network \| GIDEON (gideononline.com)](https://www.gideononline.com/) |
| 20 | South Sudan | Abyei District (South Kordufan) | 2009 | 12/06/2009 | 7 | 4 | 57.1 | no | yes | ^60^ |
| 21 | Mauritania | Arafat, Nouadhibou, Aleg | 2010 |  | 3 |  | 0.0 | -- | -- | Gideon online  [Global Infectious Diseases and Epidemiology Network \| GIDEON (gideononline.com)](https://www.gideononline.com/) |
| 22 | Namibia | Karas | 2010 | 7/1/2010 | 1 | 0 | 0.0 | yes(south Africa) | yes | ^9^ |
| 23 | South Africa | Northern Cape (Petrusburg, Rouxville) | 2010 | 01/01/2011 | 3 | -- | 0.0 | no | yes | International Society for Infectious Diseases, .ProMED  Archive  number: 20110101.0012  [Promed Post - ProMED-mail (promedmail.org)](https://promedmail.org/promed-post/?id=20110101.0012) |
| 24 | Mauritania | Riyad, Maghtaa Lahjar Aleg | 2011 | -- | 3 | -- | 0.0 | -- | -- | -- |
| 25 | Uganda | multiple (Agogo district, Kampala City) | 2013 | 16/08/2013 | 6 | 3 | 50.0 | yes (south soudan) | -- | ^61^ |
| 26 | South Africa | Bloemfontein | 2013 | 15/01/2013 | 2 | 0 | 0.0 | no | yes | International Society for Infectious Diseases, .ProMED  Archive  number: 20130115.1499675  [Promed Post - ProMED-mail (promedmail.org)](https://promedmail.org/promed-post/?id=20130115.1499675) |
| 27 | South Africa | Free state province, North West, Mpumalanga (Kalkfontien) | 2013 | 05/01/2013 | 1 | 0 | 0.0 | no | yes | International Society for Infectious Diseases, .ProMED  Archive  number: 20130115.1499675  [Promed Post - ProMED-mail (promedmail.org)](https://promedmail.org/promed-post/?id=20130115.1499675) |
| 28 | South Africa | Northern Cape province (Upington) | 2014 | 12/09/2014 | 1 | 1 | 100.0 | yes(Namibia) | yes | International Society for Infectious Diseases, .ProMED  Archive  number: 20140919.2788764  [Promed Post - ProMED-mail (promedmail.org)](https://promedmail.org/promed-post/?id=20140919.2788764) |
| 29 | Uganda | Nakaseke | 2015 | 11/06/2015 | 1 | 0 | 0.0 | no |  | ^61^ |
| 30 | Mauritania | Tintane | 2015 |  | 1 |  | 0.0 |  |  | ^62^ |
| 31 | Senegal | Ndiawdoune (Saint Louis) | 2015 | 10/11/2015 | 1 | 0 | 0.0 | no | yes | International Society for Infectious Diseases, .ProMED  Archive  number: 20151111.3784725  [Promed Post - ProMED-mail (promedmail.org)](https://promedmail.org/promed-post/?id=20151111.3784725) |
| 32 | Mauritania | Boutilimit, Nouakchott, Haye Sakin & Arafat Village in Mohammedia, Boutilimit Prefecture | 2017 | 24/08/2017 | 6 | 0 | 0.0 | yes (in Senegal) | yes | International Society for Infectious Diseases, .ProMED  Archive  number: 20170726.5205146  [Promed Post - ProMED-mail (promedmail.org)](https://promedmail.org/promed-post/?id=20170726.5205146) |
| 33 | Namibia | Gobabis District,Uukwandongo village in the Okahao district of the Omusati region, and Okongoua village, Omaheke region | 2017 | 22/02/2017 | 2 | 1 | 50.0 | no | yes | International Society for Infectious Diseases, .ProMED  Archive  number: 20170812.5244149  [Promed Post - ProMED-mail (promedmail.org)](https://promedmail.org/promed-post/?id=20170812.5244149) |
| 34 | South Africa | Western Cape province (Mamre) | 2017 | 06/01/2017 | 1 | 0 | 0.0 | no | yes | International Society for Infectious Diseases, .ProMED  Archive  number: 20170113.4762405  [Promed Post - ProMED-mail (promedmail.org)](https://promedmail.org/promed-post/?id=20170113.4762405) |
| 35 | South Africa | Northern Cape province (Van Wysksvlei) | 2017 | 03/01/2017 | 1 | 1 | 100.0 | no | yes | International Society for Infectious Diseases, .ProMED  Archive  number: 20170203.4812066  [Promed Post - ProMED-mail (promedmail.org)](https://promedmail.org/promed-post/?id=20170203.4812066) |
| 36 | Senegal | Fatick district | 2017 | 29/06/2017 | 1 | 0 | 0.0 | no | yes | International Society for Infectious Diseases, .ProMED  Archive  number: 20170726.5205146  [Promed Post - ProMED-mail (promedmail.org)](https://promedmail.org/promed-post/?id=20170726.5205146) |
| 37 | Uganda | northern region | 2018 | 17/07/2018 | 42 | -- | 0.0 | yes (DRC, Rwanda) |  | International Society for Infectious Diseases, .ProMED  Archive  number: 20180806.5949636 |
| 38 | Mauritania | Ould Yengé commune, Guidimaka region | 2018 | 24/04/2018 | 1 | 0 | 0.0 | no | yes | International Society for Infectious Diseases, .ProMED  Archive  number: 20180502.5778839  [Promed Post - ProMED-mail (promedmail.org)](https://promedmail.org/promed-post/?id=20180502.5778839) |
| 39 | Namibia | Okalongo in Omusati region, Gobabis, Omaheke | 2018 | 09/02/2018 | 1 | 0 | 0.0 | no | yes | International Society for Infectious Diseases, .ProMED  Archive  number: 20180209.5615643  [Promed Post - ProMED-mail (promedmail.org)](https://promedmail.org/promed-post/?id=20180209.5615643) |
| 40 | South Sudan | Multiple (Eastern lake state) | 2018 | 17/01/2018 | 60 | 4 | 6.7 | yes (Uganda) | yes | International Society for Infectious Diseases, .ProMED  Archive  number: 20180117.5563295  [Promed Post - ProMED-mail (promedmail.org)](https://promedmail.org/promed-post/?id=20180117.5563295) |
| 41 | Mauritania | Hodh Elgharbi | 2019 | 17/06/2019 | 2 | 0 | 0.0 | no | yes | ^63^ |
| 42 | Namibia | Multiple locations (oshikoto region, olukonda Constituency, Engela district, Oshana region ) | 2019 | 06/05/2019 | 7 | 1 | 14.3 | no | yes | International Society for Infectious Diseases, .ProMED  Archive  number: 20190518.6475254  [Promed Post - ProMED-mail (promedmail.org)](https://promedmail.org/promed-post/?id=20190518.6475254) |
| 43 | South Africa | Northern Cape province | 2019 | 24/03/2019 | 1 | 0 | 0.0 | no | yes | International Society for Infectious Diseases, .ProMED  Archive  number: 20190330.6394622  [Promed Post - ProMED-mail (promedmail.org)](https://promedmail.org/promed-post/?id=20190330.6394622) |
| 44 | South Africa | North West province | 2019 | 29/04/2019 | 1 | 0 | 0.0 | no | yes | International Society for Infectious Diseases, .ProMED  Archive  number: 20190511.6462345  [Promed Post - ProMED-mail (promedmail.org)](https://promedmail.org/promed-post/?id=20190511.6462345) |
| 45 | Senegal | Matam region (Douga village) bokidiawé | 2019 | 04/09/2019 | 1 | 0 | 0.0 | no | yes | ^64^ |
| 46 | Mali | mopti | 2020 | 02/01/2020 | 20 | 7 | 35.0 | no | yes (cattle, human) | ^65^ |
| 47 | South Africa | North West province | 2020 | 08/02/2020 | 1 | 0 | 0.0 | no | yes | International Society for Infectious Diseases, .ProMED  Archive  number: 20200215.6980424  [Promed Post - ProMED-mail (promedmail.org)](https://promedmail.org/promed-post/?id=20200215.6980424) |
| 48 | Senegal | Pikine district (Dakar) | 2020 | 17/08/2020 | 1 | 0 | 0.0 | no | yes | International Society for Infectious Diseases, .ProMED  Archive  number: 20200823.7701444  [Promed Post - ProMED-mail (promedmail.org)](https://promedmail.org/promed-post/?id=20200823.7701444) |
| 49 | Uganda | western (kikuube) | 2021 | 28/04/2021 | 2 | 0 | 0.0 | no | -- | International Society for Infectious Diseases, .ProMED  Archive  number: 20210508.8347347  [Promed Post - ProMED-mail (promedmail.org)](https://promedmail.org/promed-post/?id=20210508.8347347) |
| 50 | South Africa | Namaqualand Flower Route, Northern Cape Province | 2021 | --- | 1 | 0 | 0.0 | --- | -- | ^66^ |
| 51 | Senegal | Podor (Saint Louis region) | 2022 | 07/08/2022 | 2 | 1 | 50.0 | yes (Mauritania) | yes | ^67^ |
| 52 | Sudan | Darfour | 2015-2016 |  | 7 | 1 | 14.3 | no | yes | ^68^ |
| 53 | Uganda | central region (Nakaseke, Luweero, kiboga) | 2017-2018 | 20/08/2017 | 7 | 2 | 28.6 | no |  | ^69^ |
| 54 | South Africa | Transvaal | 1981 | -- | -- | -- | -- | -- |  | International Society for Infectious Diseases, .ProMED  Archive  number: 20210508.8347347  [Promed Post - ProMED-mail (promedmail.org)](https://promedmail.org/promed-post/?id=20210508.8347347) |

**Table 4: – Socio-ecological variables description, hypotheses and data source**

| **Variable** | **Description and sources of the covariates** | |  | **Association with the outcome of interest: CCHF outbreak** | |
| --- | --- | --- | --- | --- | --- |
|  | **Description** | **Data Source** |  | **Hypotheses and relation with CCHF infection** | **References** |
| Accessibility to Cities | This layer shows the accessibility to cities for the year 2015 | Weiss *et al.,* 2018  ^70^ |  | Goods and services are heavily concentrated in large cities, and their accessibility is an indicator of the level of development and inequality. CCHF outbreaks can be easier to detect in areas with better access to cities where the healthcare and surveillance infrastructures are more robust. | Diuk-Wasser *et al*.,2021 ^51^ |
| Mammals Species Richness | This layer shows the distribution of mammal species at 30 arc-second (~1 km) resolutions and was created by the IUCN in collaboration with the CIESIN and Columbia University | International Union for Conservation of Nature (IUCN) ^71^ |  | Mammals are part of the mandatory ectoparasitic cycle of the ticks and the CCHF virus. The diversity and abundance of mammals contribute to the maintenance and spread of CCHF. | Sara Baz-Flores *et al.,* 2024^72^ |
| Aridity Index | The layer is based on the relationship between precipitation and evapotranspiration and depicts the moisture availability for potential growth of vegetation. | Trabucco & Zomer., 2018  ^73^ |  | The aridity index is linked to the vegetation of a given area. Ticks presence depends type of vegetation | El Ghassem *et al.,* 2023^74^  Fanelli *et al.,* 2023 ^75^ |
| Digital Elevation Model and Slope | We used the Shuttle Radar Topography Mission Digital Elevation Model (SRTM-DEM) to show variation in altitude and slope in the region. | Farr *et al.*, 2007 ^76^ |  | The elevation is a condition for vegetation type, climatic conditions that can be suitable for ticks proliferation. | Estrada-Peña, De la Fuente, 2014 ^47^  Aker *et al .,* 2015 ^27^ |
| Global Land Cover - SHARE (GLC-SHARE) | These layers were used to capture the variation in land cover land use of the region. | Latham *et al.,* 2014 ^37^ |  | The relationship between pattern of vegetation, land use and the tick habitat suitability are widely demonstrated in many ecological niches modelling studies. | Messina *et al.,* 2015 ^29^  Messina *et al.,* 2023^45^  Chanda *et al*., 2023 ^31^ |
| Population Density | WorldPop estimation of 2020 population was used in this study | WorldPop (2018) ^38^ |  | The human population density is a function of the “attractiveness” of the area. It is also a function of the vegetation: drylands with higher human populations and more livestock are at higher risk of CCHF outbreaks than more arid, less populated areas. | Ahmadkhani *et al.,* 2017 ^24^  Telford *et al.,* 2023 ^48^ |
| Livestock Spatial Distribution | The layers provide livestock densities in each pixel for each livestock species and at a spatial resolution of 0.083333 decimal degrees (approximately 10 km at the equator) | Gilbert *et al.,* 2018 ^77^ |  | Considering the key role of livestock in the life cycle of ticks and the risk of contamination among professionals who interact animals, the density and spatial distribution of livestock could be a predictive factor in the onset of the disease. | Bente *et al*., 2013 ^3^  Estrada-Peña, De la Fuente, 2014 ^47^ |
| Woodcover | This layer showed the spatial variation of woody and herbaceous vegetation in Sub-Sahara region. | Kahiu & Hanan, 2018 ^78^ |  | The type of vegetation in a given area is associated with the presence and proliferation of ticks likely to carry the virus. | Messina *et al.,* 2015 ^29^  Messina *et al.,* 2023^45^  Vescio *et al*., 2012 ^26^  Telford *et al.,* 2023 ^48^ |
| Climatic Data | Bioclimatic variables from www.worldclim.org were used to capture the climatic effect | Fick and Hijmans, 2017 ^36^ |  | The emergence and propagation of the CCHF virus are significantly influenced by climatic conditions and climate change over time. Various factors, including temperature, precipitation, and humidity, affect the distribution and activity of tick vectors that transmit the CCHF virus | Estrada-Peña, De la Fuente, 2014 ^47^  Nili *et al*., 2020 ^30^  Fanelli et *al.,* ^75^ |
| Distance to Parks | The layer contains polygons of national parks and game reserves which were used to compute distance to parks | UNEP-WCMC and IUCN 2019 ^40^ |  | Parks and natural reserves and green spaces within and surrounding urban settings are places of contact between humans and ticks. Their accessibility to the public increases the potential risk of tick bites and associated infection such as CCHF. | Sara Baz-Flores *et al.,* 2024^72^  El Ghassem *et al.,* 2023^71^ |
| Births Attended by Specialist | This data shows the percentage of births attended by a specialist | World Bank ^39^ |  | The proportion of births attended by specialists is a reliable indicator of the quality and accessibility of healthcare services and surveillance system in a country. In the context of the SSA region, the ability to detect epidemics may be limited, leading to underreporting of CCHF cases. This covariate helps account for this situation. | Temur *et al .,* 2021^9^  Bair *et al.,* 2019^79^ |
